# Resilience to substance use disorder following childhood maltreatment: association with peripheral biomarkers of endocannabinoid function and neural indices of emotion regulation

**DOI:** 10.1038/s41380-023-02033-y

**Published:** 2023-04-12

**Authors:** Irene Perini, Leah M. Mayo, Andrea J. Capusan, Elisabeth R. Paul, Adam Yngve, Robin Kampe, Emelie Gauffin, Raegan Mazurka, Bijar Ghafouri, Niclas Stensson, Anna Asratian, J. Paul Hamilton, Åsa Kastbom, Per A. Gustafsson, Markus Heilig

**Affiliations:** 1 Center for Social and Affective Neuroscience, Department of Biomedical and Clinical Sciences, Linköping University, Linköping, Sweden; 2 Center for Medical Image Science and Visualization (CMIV), Linköping, Sweden; 3 Department of Psychiatry, Linköping University Hospital, Linköping, Sweden; 4 Department of Biomedical and Clinical Sciences, Linköping University, Linköping, Sweden; 5 Pain and Rehabilitation Centre, Department of Health, Medicine and Caring Sciences, Linköping University, Linköping, Sweden; 6 Department of Biological and Medical Psychology University of Bergen, Bergen, Norway; 7 Department of Child and Adolescent Psychiatry, Linköping University, Linköping, Sweden

**Keywords:** Addiction, Neuroscience

## Abstract

Childhood maltreatment (CM) is a risk factor for substance use disorders (SUD) in adulthood. Understanding the mechanisms by which people are susceptible or resilient to developing SUD after exposure to CM is important for improving intervention. This case-control study investigated the impact of prospectively assessed CM on biomarkers of endocannabinoid function and emotion regulation in relation to the susceptibility or resilience to developing SUD. Four groups were defined across the dimensions of CM and lifetime SUD (*N* = 101 in total). After screening, participants completed two experimental sessions on separate days, aimed at assessing the behavioral, physiological, and neural mechanisms involved in emotion regulation. In the first session, participants engaged in tasks assessing biochemical (i.e., cortisol, endocannabinoids), behavioral, and psychophysiological indices of stress and affective reactivity. During the second session, the behavioral and brain mechanisms associated with emotion regulation and negative affect were investigated using magnetic resonance imaging. CM-exposed adults who did not develop SUD, operationally defined as resilient to developing SUD, had higher peripheral levels of the endocannabinoid anandamide at baseline and during stress exposure, compared to controls. Similarly, this group had increased activity in salience and emotion regulation regions in task-based measures of emotion regulation compared to controls, and CM-exposed adults with lifetime SUD. At rest, the resilient group also showed significantly greater negative connectivity between ventromedial prefrontal cortex and anterior insula compared to controls and CM-exposed adults with lifetime SUD. Collectively, these peripheral and central findings point to mechanisms of potential resilience to developing SUD after documented CM exposure.

## Introduction

Childhood maltreatment (CM) is associated with a wide range of adverse physical and mental health outcomes [1]. We recently reported that the risk of developing substance use disorders (SUD), including alcohol use disorder (AUD), in individuals with prospectively documented exposure to severe CM remained 3-fold elevated after controlling for familial confounding [2]. The impact of CM is shaped by complex genetic, environmental, and cognitive factor [1]. The mechanisms by which some individuals are susceptible or resilient to developing SUD after exposure to CM are still not understood.

Meta-analytic evidence has shown an association between CM and deficits in emotion regulation [3, 4], a psychological process that is crucially involved in the management of stress, a key trigger of relapse in SUD. Emotional regulation is a complex clinical construct, originally defined as: “an ongoing process of the individual’s emotion patterns in relation to moment-by-moment contextual demands” [5]. At the brain level, emotional regulation relies on the integration of several basic and high-order cognitive processes, including motivational salience, attention, and emotion processing, and engages cortical and subcortical regions, including medial prefrontal cortex (mPFC) and amygdala [4]. The disruption of typical neurobiological development by exposure to CM, together with genetic vulnerability, can challenge the acquisition of adaptive emotion regulation strategies. In humans, the type of CM, timepoint, and duration of exposure, and psychiatric condition at testing critically affect outcomes, but evidence generally supports altered corticolimbic and salience processing in individuals exposed to CM [6].

Corticolimbic circuitry integral to emotion regulation capabilities is modulated by the endocannabinoid (eCB) system. The eCBs anandamide (AEA) and 2-arachidonoylglycerol (2-AG) are key mediators of stress and emotion processing [7–10]. Evidence from animal studies has shown that eCB function within the amygdala is critical for regulation of stress and threat responding, which is constrained by inputs from prefrontal cortical regions [11, 12]. The eCB system undergoes extensive restructuring during childhood and adolescence, including dynamic fluctuations in cannabinoid receptors, ligands, and catabolic enzymes [13, 14]. Perturbations of this process can lead to sustained effects on eCB signalling and gene expression that persist into adulthood, impacting stress and emotion regulatory processes[13–22]. Thus, the eCB system plays a critical role in emotion regulation that may be impacted by early life stressors such as CM.

Altogether, the widespread brain changes in CM-exposed individuals suggest that altered corticolimbic interactions, potentially influenced by eCB signalling, can impact emotion regulation processes. Whether these changes reflect potentially adaptive mechanisms specific to CM exposure can be difficult to disentangle unless resilient and susceptible groups are directly compared. Moreover, the use of retrospective assessments can complicate matters due to poor agreement between prospective and retrospective assessments [23], and since current psychopathology in adulthood influences retrospective reports of CM [24]. Here, we searched for features that may distinguish individuals that are resilient or susceptible to developing SUD on the basis of prospectively documented CM exposure. Specifically, we assessed peripheral levels of eCB ligands AEA and 2-AG at baseline and in response to an experimental stressor, in addition to behavioral and physiological measures of stress and affective processing. To investigate central changes in emotion regulation, we assessed brain activity during the emotional conflict task [25] and resting-state. We predicted that altered brain and eCB function may contribute to greater emotion regulation impairments, which would be particularly notable in the CM-exposed individuals who subsequently developed an SUD.

## Material and Methods

### Study overview

This study consisted of three visits: one screening visit, a second behavioral laboratory session, and a final magnetic resonance imaging (MRI) session. During screening, participants were evaluated for eligibility and upon inclusion, blood samples were collected for genotyping (Supplementary Methods). In the first laboratory session, blood samples and psychophysiological recordings were collected while participants completed a series of behavioral tasks assessing stress and emotional reactivity. In the final visit, the MRI session, one anatomical, one resting-state and three task-based scans were collected. Task-based measures aimed at assessing emotion regulation, and processing of negative affect (Supplementary Methods and Results) and alcohol-related stimuli (not included in the current manuscript). Participants completed breath and urine screens for alcohol and drugs prior to laboratory sessions. All behavioral data were analyzed using the Statistical Package for Social Sciences (SPSS) software version 28.0.1.0 and graphs were created in Prism 9. All analyses were two-tailed.

### Participants

Participants were recruited between March 2017 and July 2020, at Linköping University. A total of 101 participants were included in the study, divided into four groups across the dimensions of CM and SUD. The first group had both CM and lifetime SUD (CM + SUD, *N* = 28); the second group, operationally defined as resilient, had CM without lifetime SUD (CM only, *N* = 24); the third group consisted of a healthy control group with neither CM nor lifetime SUD (control, *N* = 24); finally, the fourth group consisted of a clinical control group with lifetime SUD but no documented CM (SUD only, *N* = 25).

All CM exposed participants (CM only and CM + SUD) consisted of former patients in a specialized treatment unit [2] for children and adolescents exposed to physical and/ or sexual abuse and/ or severe neglect referred by the child protective services. The Swedish personal identification number allowed the identification and long-term follow-up of these former CM treatment unit patients, now young adults, using the regional health care register for Östergötland County, Sweden [26] (*N* = 470). Sixty-five former CM treatment unit patients with both documented CM exposure and documented contact with SUD clinics were eligible. For each of these participants, we identified sex/age-matched CM exposed eligible individuals with no lifetime SUD (*N* = 140), and sex/age matched individuals with lifetime SUD but with no recorded CM exposure (*N* = 106). Controls with lifetime SUD but no documented CM were recruited using the regional health care register and through advertisements from addiction clinics in the Region of Östergötland. Sex and age-matched healthy controls with no documented SUD or CM were recruited through advertising among students at Linköping University and social media. Participants meeting eligibility criteria were contacted by phone and invited to participate in a screening session described in detail in Supplementary Methods. A CONSORT flow-chart of study participants is presented in Figure S1. The study was approved by the Regional Ethics Review Board in Linköping, Sweden (Dnr 2015/256-31, and 2017/41-32).

### Behavioral session

#### Overview

Upon arrival at the lab, participants were fitted with an intravenous catheter for blood sample collection and prepared for psychophysiological recordings via application of facial electromyography (EMG) recording electrodes and disposable electrodes to measure electrocardiography (ECG) and electrodermal (EDA) activity (i.e. skin conductance) as previously described [27, 28]. Participants completed a series of behavioral tasks assessing emotion and stress reactivity [27, 29]. Blood samples were collected throughout the session (i.e., at baseline, prior to stress exposure, immediately following stress, and during recovery from stress) to measure baseline and stress-induced changes in peripheral endocannabinoids and cortisol. See Supplementary Methods for detailed descriptions.

In all analyses, between-subjects effects of CM (yes/no) and SUD (yes/no) and CM x SUD interactions were included. Significant interactions were followed up with Bonferroni-corrected between-group comparisons. For all analyses, significance was set at *P* < 0.05, and reported p-values were corrected for multiple comparisons.

#### Endocannabinoid analysis

The eCBs (AEA and 2-AG) and *N*-acylethanolamines (NAEs), oleoylethanolamide (OEA) and palmitoylethanolamide (PEA) were extracted and analyzed using liquid chromatography tandem mass spectrometry (LC-MS/MS), as previously published [30] (Supplementary Methods). Endocannabinoid values were log-transformed due to non-normality of the distribution; these transformed values were used in all subsequent analyses. Baseline differences in eCBs were analyzed as the dependent variable in a one-way ANOVA. Endocannabinoid responses to stress were analyzed using a repeated measure (RM)-ANOVA with time as a within-subjects factor.

#### Affective images

The affective image task [31] was completed before and after stress exposure. It consisted of positive, neutral, and negative images selected from the International Affective Picture System (IAPS [32]). Participants viewed a single image at a time, and then rated it on valence and arousal. Facial EMG responses were quantified as the mean EMG amplitude during the 6 sec image presentation compared to the preceding 1 sec baseline. Data were analyzed using RM-ANOVA with stimulus type (positive, neutral, negative) as the within-subject factor for each muscle (corrugator, zygomatic) and self-report rating (valence, arousal).

#### Acute stress reactivity

The Maastricht Acute Stress Test (MAST) is a 10 min task consisting of alternating hand immersion in ice-cold water and mental arithmetic trials with negative socio-evaluative feedback [33]. Blood samples were collected via the indwelling catheter in the arm not submerged during the task [27]. See Supplementary Methods for details on the MAST task and blood data collection.

### Magnetic resonance imaging session

#### Emotional conflict task

Participants performed the emotional conflict task [25] in the MRI scanner. A series of 148 consecutive pictures of fearful or happy facial expressions were presented with a word superimposed on the face. The words “fear” and “happy” were used and could be either congruent or incongruent with the facial expressions. Pictures were presented for 1000 ms, with jittered fixation intervals (3000–5000 ms). Participants were asked to identify the two emotions of the face, while ignoring the word, by pressing with their index and middle fingers. In the original version of the task [25], the authors found that the decrease in performance after exposure to the first incongruent trial, reflected by increased reaction times and worse accuracy, was attenuated when a second incongruent trial was presented. The observed behavioral effect led to the hypothesis that the first incongruent trial would reflect conflict monitoring, whereas the second incongruent trial would reflect conflict resolution processes. To exclude potential differences in motor reactivity between fingers, response fingers were counterbalanced across emotion type. Sex and emotion depicted on the pictures were counterbalanced. Images were presented using Presentation Software version 17.2 (Neurobehavioral Systems, Inc. Berkley, San Francisco, USA).

Accuracy, and reaction times (RTs) were extracted for the behavioral analysis. Scores ± 2 SD from the group means were removed, and a cut-off limit of 50% was used for overall accuracy. We used two linear mixed effects (LME) models that considered the full-factorial nature of group recruitment strategy and included CM and SUD as factors. For both analyses, subject was included as random effect variable and sex (male/female) was included as a binary covariate.

The first model aimed to identify potential replication of the original study [25] (Supplementary Methods). However, the canonical previous x current trial interaction for RTs and accuracy scores in incongruent trials did not replicate. Therefore, in the second LME model, we categorized behavioral scores depending only on whether the current trial was congruent or incongruent. As a design-driven add-on, we also included emotion as a within-subject factor. Thus, a 2x2x2x2 LME analysis was performed with the following factors: CM (yes/no) X SUD (yes/no) X trial (congruent/incongruent) X emotion (fear/happy).

#### MRI data preprocessing and analysis

MRI data acquisition, preprocessing, and analyses information is presented in detail in the Supplementary Methods. Preprocessing and statistical analyses were performed with the Analysis of Functional Neuro Images (AFNI) software v18.3.16 [34]. Results were thresholded at a whole-brain, gray matter level, using a per-voxel *P* = 0.002, and multiple comparison corrected at *alpha* = 0.05 [35], clustering method 2. Beta coefficients from significant interactions were compared between groups using a one-way MANCOVA with psychotropic medication use as covariate, and post-hoc comparisons were corrected with Tukey’s test.

For the emotional conflict task, four regressors of interest, based on trial and emotion type were created and modelled across the 1000 ms interval corresponding to picture presentation. An additional regressor modelling button presses was included in the regression. For group analysis, a 2x2x2x2 linear mixed-effects (LME) model was performed at whole-brain, gray matter, voxel-wise level using the AFNI function 3dLME [36]. Factors were the same as for the behavioral analysis. Subject was included as random effect.

Resting-state data were preprocessed according to current AFNI recommendations (see Example 11 in afni_proc.py and Supplementary Methods). Three seeds were used, defined based on the emotional conflict task results. Seed to whole brain connectivity analyses were performed by entering seed time course as predictor in a regression analysis, using 3dDeconvolve. For group analysis, resulting beta coefficients for each seed location were entered in a 2x2 LME analysis with factors CM (yes/no) and SUD (yes/no) using 3dLME [36].

## Results

### Participants

Participants’ demographics are presented in Table 1. The sociodemographic between-group differences presented in Table 1 are driven by the control group. No significant differences in these variables were found between the CM + SUD, SUD, and CM only groups (all *Ps* > 0.05). Thirteen participants in the CM + SUD group, and 10 in the SUD only group had a MINI [37] diagnosis of ongoing (last 12 months) SUD, including AUD. In addition, 1 participant in the CM + SUD group and 5 participants in the SUD only group had positive urine drug screen tests at all visits for amphetamine, tetrahydrocannabinol (THC), opioids, or benzodiazepines, indicating ongoing SUD. Sensitivity analyses, conducted by removal of participants with positive drug screening at experimental visits, and that affected main findings, are reported in the results.

**Table 1:** Demographic, clinical, and psychological characteristics of the population.

	CM with SUD	CM only	SUD only	Control	*p*-value
	*N* = 28	*N* = 24	*N* = 25	*N* = 24	
Sex: Female	15 (54%)	17 (71%)	12 (48%)	13 (54%)	0.41
Age	28.9 (3.5)	28.9 (3.9)	27.5 (3.3)	28.3 (5.2)	0.56
Education					<0.001
Elementary school	5 (18%)	1 (4%)	6 (24%)	0 (0%)	
Vocational education	14 (50%)	13 (54%)	11 (44%)	1 (4%)	
High school	4 (14%)	1 (4%)	4 (16%)	2 (8%)	
University	4 (14%)	7 (29%)	4 (16%)	21 (88%)	
Born in Sweden	22 (79%)	15 (63%)	18 (72%)	18 (75%)	0.38
Current psychiatric diagnosis (MINI)^1^	23 (82%)	9 (38%)	20 (80%)	1 (4%)	<0.001
Psychotropic medication^2^	13 (46%)	4 (17%)	15 (60%)	3 (13%)	<0.001
Current SUD/AUD (MINI)	13 (46%)	0 (0%)	10 (40%)	0 (0%)	<0.001
Current SUD (MINI)	6 (21%)	0 (0%)	3 (11%)	0 (0%)	=0.003
Current AUD (MINI)	11 (39%)	0 (0%)	8 (28%)	0 (0%)	<0.001
AUDIT	8.2 (5.7)	4.3 (2.6)	6.3 (6.2)	3.9 (3.4)	0.005
DUDIT	3.8 (7.4)	0.1 (0.4)	6.2 (7.9)	.0 (.0)	<0.001
CPRS total scores	15.5 (9.0)	9.4 (7.1)	12.3 (7.4)	4.3 (3.8)	<0.001
CPRS Depression scores (MADRS)	8.1 (4.7)	4.0 (3.7)	5.7 (4.1)	1.7 (1.8)	<0.001
CPRS_Anxiety	7.9 (4.9)	5.8 (3.9)	7.0 (3.6)	3.0 (2.4)	<0.001
DERS total Scores	43.1 (16.4)	34.8 (14.2)	40.6 (15.1)	25.9 (7.3)	<0.001
CTQ Scores					
Total score	50.3 (19.8)	51.5 (18.9)	42.6 (16.1)	28.3 (3.9)	<0.001
Physical abuse	9.4 (4.8)	8.7 (3.9)	6.6 (2.9)	5.1 (.3)	<0.001
Sexual abuse	8.4 (5.5)	9.8 (6.6)	5.2 (.7)	5.0 (.0)	<0.001
Emotional abuse	12.1 (5.7)	11.6 (5.4)	10.7 (5.4)	5.7 (1.0)	<0.001
Physical neglect	8.3 (3.0)	8.9 (4.4)	8.4 (4.6)	5.3 (.7)	0.003
Emotional neglect	12.1 (5.7)	12.5 (4.7)	11.6 (5.5)	7.2 (2.9)	<0.001
ADHD TOTAL scores (MINI)	7.6 (5.1)	3.9 (3.6)	7.6 (5.5)	1.6 (1.7)	<0.001
MRI session	*N* = 25	*N* = 22	*N* = 23	*N* = 24	
Task Behavior^3^ - Accuracy (RT)	*N* = 22 (24)	*N* = 22 (22)	N = 20 (21)	N = 23 (24)	
Task-based fMRI analysis^3^	*N* = 25	*N* = 22	*N* = 22	*N* = 24	
Task-based fMRI analysis^4^	*N* = 25	*N* = 22	*N* = 21	*N* = 24	
Resting state fMRI analysis	*N* = 23	*N* = 22	*N* = 23	*N* = 23	

Data are presented as mean (SD) for continuous measures, and n (%) for categorical measures. Between-group differences in sociodemographic scores were driven by the healthy controls group. ^1^ongoing psychiatric diagnosis according to MINI screening; ^2^stable standard doses for at least three months of common psychotropic medications; ^3^Emotional Conflict Task; ^4^Negative Affect Picture Task; *SUD* substance use disorder, current diagnosis according to MINI interview, *AUDIT* Alcohol Use Disorders Identification Test, *DUDIT* Drug Use Disorders Identification Test, *CPRS* Comprehensive Psychopathological Rating Scale, self-report, *DERS* Difficulties in Emotion Regulation Scale, *NEO* Neuroticisms=NEO Five-Factor Inventory, Neuroticism; *CTQ* childhood trauma questionnaire, *ADHD* total number of symptoms from the inattentive and hyperactive impulsive subscales.

### Behavioral Session

#### Endocannabinoids and genotyping

**Fig. 1:**
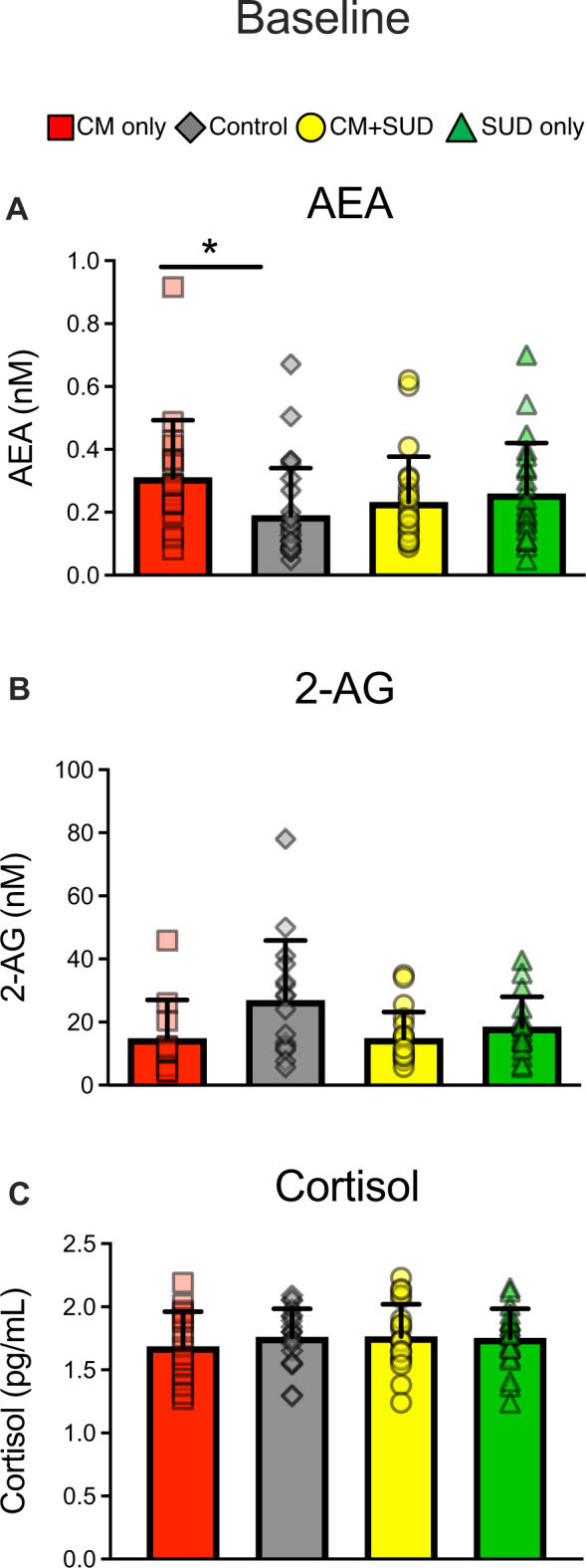
Consequences of Childhood Maltreatment (CM) and Substance Use Disorder (SUD) histories on baseline endocannabinoids and cortisol. At baseline, the CM only group had significant higher levels of anandamide (AEA) than controls **(A**, *P* = 0.015), while both CM groups had lower peripheral levels of 2-AG (**B**; effect of CM, *P* = 0.011). There were no effects of CM or SUD on baseline cortisol levels (**C**). *P < 0.05 for group comparison (vs. controls). Note that values shown are raw data, but analysis were conducted on log transformed data due to non-normality of the distribution.

At baseline, eCB levels differed according to CM exposure. AEA levels were significantly different between groups (F_3,82_ = 3.37, *P* = 0.023, partial η^2^ = 0.11), with a main effect of CM (F_1,82_ = 4.02, *P* = 0.048, partial η^2^ = 0.047) and a CM x SUD interaction (F_1,82_ = 6.32, *P* = 0.014, partial η^2^ = 0.073; Fig. 1A). Follow-up tests revealed that the CM only group, operationally defined as a resilient group, had significantly higher levels of AEA than the control group (*P* = 0.015). Baseline 2-AG levels were significantly different between groups (F_3, 60_ = 2.81, *P* = 0.047, partial η^2^ = 0.12), with a main effect of CM (F_1,.48_ = 6.89, *P* = 0.011, partial η^2^ = 0.10; Fig. 1B). Post-hoc follow-up tests showed that 2-AG levels were not significantly lower in either the CM only (*P* = 0.061) and CM + SUD (*P* = 0.097) groups as compared to controls. Baseline levels of cortisol (*P* = 0.72; Fig. 1C), OEA (*P* = 0.75), and PEA (*P* = 0.69) did not differ between groups.

**Fig. 2:**
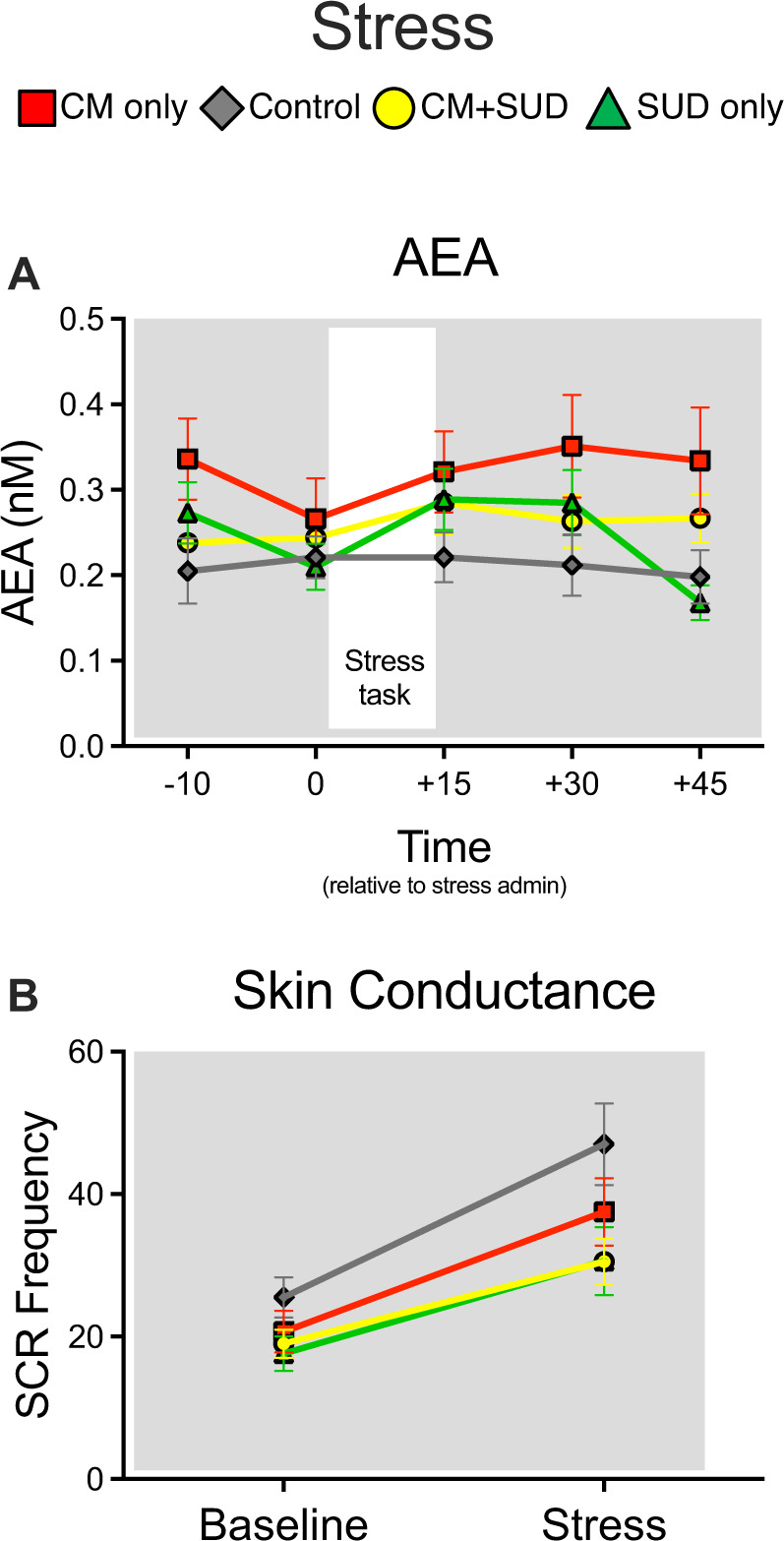
Effects of stress on endocannabinoids and psychophysiological measures. Levels of AEA remained elevated in the CM only group even during stress exposure (**A**, time x CM x SUD interaction: *P* = 0.016). There was an overall effect of stress on skin conductance (*P* < 0.001) that was attenuated by SUD overall (*P* = 0.014) but did not significantly differ in either CM group (**B**). Note that values shown are raw data, but analysis were conducted on log transformed data due to non-normality of the distribution.

In the CM only group, AEA levels remained high throughout stress exposure (Fig. 2A). There was a significant within-subject effect of time (F_4,292_ = 2.84, *P* = 0.025, partial η^2^ = 0.037) and a time x CM x SUD interaction (F_4,929_ = 3.10, *P* = 0.016, partial η^2^ = 0.041) on AEA levels throughout the session, as well as a between-subject effect of CM (F_1,73_ = 6.01, *P* = 0.017, partial η^2^ = 0.076). Follow-up tests showed that the CM only group had significant higher AEA levels than the control group (*P* = .044) throughout the entire session. There was a significant effect of time on cortisol levels (F_4,292_ = 13.5, *P* < 0.001, partial η^2^ = 0.16), but no other significant effects or interactions.

Groups did not differ regarding the variation at FAAH C385A (rs324420), which has previously been shown to influence AEA levels [15] (*χ*^*2*^ (3, 88) = 2.41, *P* = 0.49).

#### Affective images

At baseline, the CM only group rated negative images as less arousing (Figure S2). There was a main effect of picture type (F_2,192_ = 56.9, *P* < 0.001, partial η^2^ = 0.37), a type x CM interaction (F_2,192_ = 5.37, *P* = 0.005, partial η^2^ = 0.057), and a type x CM x SUD interaction (F_2,192_ = 3.67, *P* = 0.027, partial η^2^ = 0.032) on arousal ratings. Follow-up tests revealed that this effect was specific to negative images (F_3,95_ = 2.79, *P* = 0.045, partial η^2^ = 0.73), with the CM only group rating negative images as less arousing than the control group (*P* = 0.039). We found no significant effects of stress on arousal ratings. See Supplementary Results for results on valence ratings and facial EMG responses.

#### Acute stress reactivity

Overall, there were limited differences in stress reactivity across groups. As expected, there was a main effect of stress on non-specific skin conductance response (SCR) frequency (F_1,91_ = 74.9, *P* < 0.001, partial η^2^ = 0.45; Fig. 2B), as well as a between-subject effect of SUD on SCR frequency (F_1,91_ = 6.30, *P* = 0.014, partial η^2^ = 0.038) such that both SUD groups had fewer SCR events irrespective of stress. Additional results are presented in the Supplementary Results.

### Magnetic resonance imaging session

#### Emotional conflict task

*Behavioral* findings. In the first LME analysis we replicated the known interference effect introduced by incongruent stimuli, evidenced by slower RTs and lower accuracy across all groups (RTs: F_1,254_ =  265, *P* < 0.001; accuracy: F_1,185_ = 109, *P* < 0.001). However, no significant interaction between the current and previous stimulus type was identified (Supplementary Methods), and this factor was therefore dropped from the analysis.

We found similar results in the second LME analysis (Supplementary Results and Fig. S3A). Both RTs and accuracy were affected by trial, with slower RTs and lower accuracy to incongruent trials (RTs: F_1,235_ = 158, *P*<0.001; accuracy: F_1,223_ = 72, *P* < 0.001). RTs were also affected by emotion, with slower RTs for fearful compared to happy faces (F_1,235_ = 19, *P* < 0.001). In addition, a trial x emotion interaction was observed, with slower RTs for congruent fearful versus congruent happy images (F_1,235_ = 6.73, *P* = 0.010). Finally, only for accuracy, a main effect of SUD was identified (F_1,84_ = 6.73, *p* = 0.011), with lower accuracy in participants with SUD (mean difference = −4% ± SEM 1.55%).

**Fig. 3:**
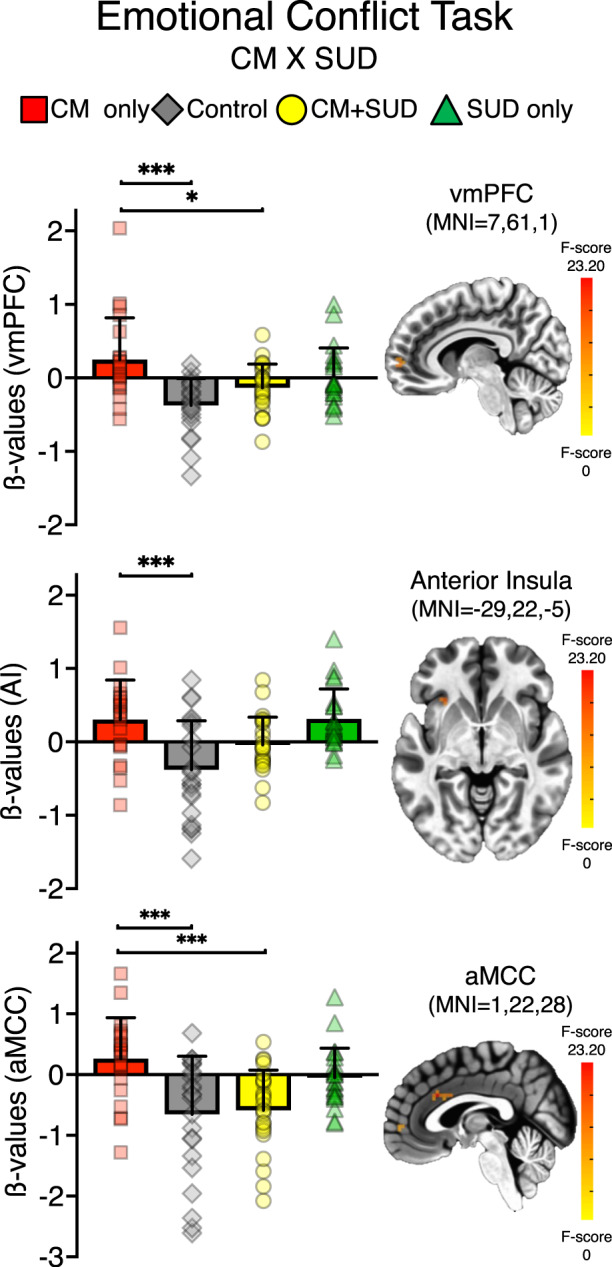
Emotional conflict task. A 2x2x2x2 linear LME model was performed at whole-brain, gray matter, voxel-wise level, with following factors: CM (yes/no) X SUD (yes/no) X Trial (conguent/incongruent) X Emotion (fear/happy). Per voxel *P* = 0.002, multiple comparison corrected at alpha = 0.05. Bar charts reflect ß-values extracted on significant clusters for the significant CM x SUD interaction, and post-hoc significance for the CM only group. *P < 0.05, **P < 0.01, ***P < 0.001.

fMRI findings. A main effect of trial was identified, with increased bilateral activity to incongruent trials in regions typically engaged by conflict processing, including anterior insula, inferior parietal lobule, and medial prefrontal cortex (Table S1, Figure S3B). A CM x SUD interaction was identified in right ventromedial prefrontal cortex (vmPFC; MNI coordinates = 7,61,1; 10 voxels), left anterior insula (AI; MNI = − 29, 22, −5; 10 voxels) and anterior midcingulate cortex (aMCC; MNI = 1, 22, 28; 15 voxels). The post-hoc analysis on extracted ß coefficients confirmed the main effect of group for vmPFC (F_3,85_ = 9.2, *P* < 0.001, partial η^2^ = 0.25), AI (F_3,85_ = 10.2, *P* < 0.001, partial η^2^ = 0.26), and MCC (F_3,85_ = 8.3, *P* < 0.001, partial η^2^ = 0.23). No significant effect of psychotropic medication was found (all *P*s > 0.05). The CM only group had increased activity compared to CM + SUD and controls across all trials (Fig. 3). Specifically, for the vmPFC cluster, the CM only group had increased activity compared to the CM + SUD group (mean difference = 0.38, *P* = 0.017) and controls (mean difference = 0.62, *P* < 0.001). For the AI cluster, the CM only had increased activity compared to the controls (mean difference = 0.68, *P* < 0.001). Finally, for the aMCC cluster, the CM only group had increased activity compared to the CM + SUD group (mean difference = 0.83, *P* < 0.001) and to controls (mean difference = 0.87, *P* < 0.001). The SUD group had increased activity compared to controls in aMCC (mean difference = 0.56, *P* = 0.03), vmPFC (mean difference = 0.39, *P* = 0.01), and AI (mean difference = 0.69, *P* < 0.001). Finally, after removal of participants with positive drug tests, AI activity was significantly increased in the SUD only group compared to the CM + SUD group (mean difference = 0.44, *P* = 0.03).

#### Resting-state

The regions identified by the CM x SUD interaction were used as seeds for the connectivity analysis of resting-state data. We found significant differences in vmPFC and AI seed-based connectivity in the CM only group compared to CM + SUD and controls.

For the vmPFC seed, a CM x SUD interaction was identified in left AI (MNI = − 32, 25, −5; 12 voxel). The post-hoc analysis on extracted ß coefficients confirmed the main effect of group (F_3,85_ = 8.6, *P* < 0.001, partial η^2^ = 0.23), and no significant effect of psychotropic medication (*P* = 0.89). A significant anticorrelation between vmPFC and AI was found in the CM only compared to the CM + SUD group (mean difference = −0.13, *P* = 0.002) and to controls (mean difference = −0.14, *P* < 0.001). The SUD only group also showed decreased vmPFC-AI connectivity compared to CM + SUD (mean difference = −0.11, *P* = 0.014) and controls (mean difference = −0.11, *P* = 0.006).

**Fig. 4:**
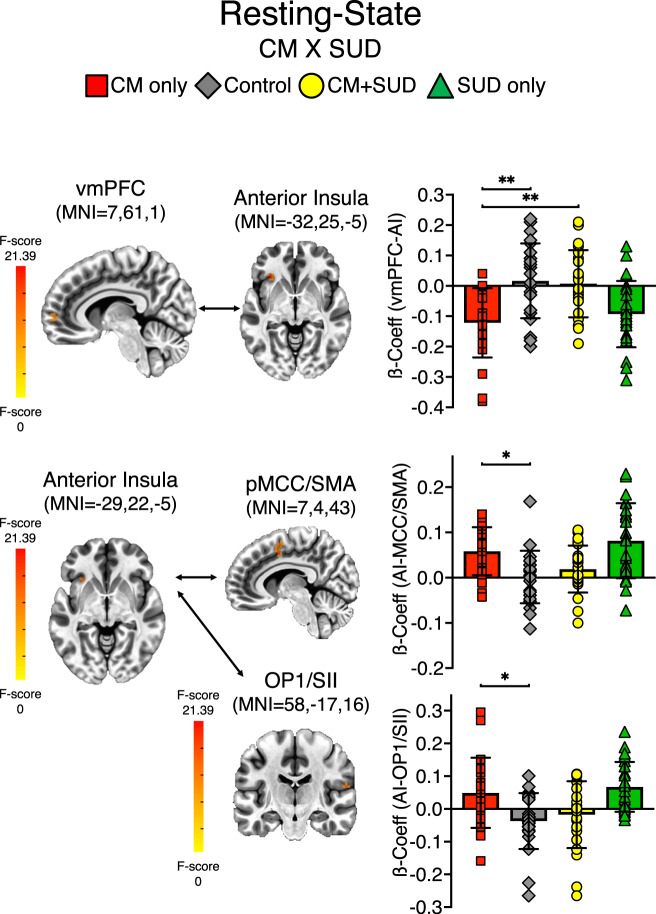
Resting-state connectivity. A 2 x 2 LME analysis was performed on beta coefficients resulting from seed-based time-course connectivity, with factors: CM (yes/no) and SUD (yes/no). Per voxel *P* = 0.002, multiple comparison corrected at alpha = 0.05. Bar charts reflect ß-Coefficients extracted on significant clusters for the significant CM x SUD interaction, and post-hoc significant difference for the CM only group. *P < 0.05, **P < 0.01, ***P < 0.001.

For the AI seed, a CM x SUD interaction was identified in the supplementary motor area (SMA) merging with posterior midcingulate cortex (pMCC) (MNI = 7, 4, 43; 20 voxels) and parietal operculum (OP1) (MNI = 58, −17,16; 13 voxels) (Fig. 4). The post-hoc analysis on extracted ß coefficients confirmed the main effect of group for connectivity between AI-MCC (F_3,85_ = 7.22, *P* < 0.001, partial η^2^ = 0.20) and AI-OP1 (F_3,85=_6.21, *P* < 0.001, partial η^2^ = 0.18). No significant effect of psychotropic medication was found (*P* > 0.4). Increased positive connectivity between AI and SMA/pMCC was found in the CM only group compared to controls (mean difference = 0.06, *P* = 0.02). Similarly, increased positive connectivity between AI and OP1 was found in the CM only group compared to controls (mean difference = 0.09, *P* = 0.015). The SUD group showed increased AI-SMA/pMCC connectivity compared to CM + SUD (mean difference = 0.06, *P* = 0.008) and controls (mean difference = 0.08, *P* < 0.001), and increased AI-OP1 connectivity compared to CM + SUD (mean difference = 0.09, *P* = 0.013) and controls (mean difference = 0.11, *P* = 0.002). Post-hoc results were not affected by removal of participants with positive drug screening at MRI visit.

## Discussion

We investigated potential mechanisms for susceptibility or resilience to developing SUD after exposure to CM, using a prospectively documented CM assessment and a factorial recruitment design. We found consistent differences between the putative resilient group (CM only) and controls across eCB and brain measures. The CM only group had increased AEA levels at baseline and during stress, compared to controls. Similarly, the CM only group had increased activity in salience and emotion regulation regions, in task-based measures of emotion regulation. In addition, a negative connectivity between vmPFC and anterior insula was found in the CM only group at rest. We speculate that the consistent differences between the CM only group and controls suggest a potential mechanism that may render these individuals particularly resilient to SUD development following CM exposure. Individuals lacking these specific features, such as the CM + SUD group, may be less well-equipped to overcome the impact of CM exposure on stress and affective processing, potentially rendering them more susceptible to SUD development.

Evidence in the literature supports altered corticolimbic and salience processing in individuals exposed to CM [6], and increased amygdala reactivity to salient emotional faces across the lifespan [38–40], indicating increased monitoring of potentially threatening social stimuli in the environment. Findings from resting-state connectivity studies suggest reduced strength of top-down control of the amygdala by medial-PFC (mPFC), portions of the cingulate including ACC and PCC, and insula [41]. Consistently, graph theory evidence shows decreased centrality in ACC, mPFC and temporal pole and increased centrality in precuneus and right anterior insula [42].

The brain findings presented in this work in salience and prefrontal regions are consistent with the literature above and might indicate adaptive coping mechanisms in the CM only group. The lack of a between-group difference in amygdala, another region implicated in salience processing, might relate to our group categorization and prospective assessment, which are not typically used in previous studies. Further studies might clarify this potential inconsistency. During affect processing, the CM only group presented with enhanced salience processing, as revealed by increased activity in AI and aMCC. This supports previous evidence showing increased responsiveness in salience processing regions to affective stimuli in individuals exposed to CM [43]. We hypothesized that the presence of this finding in the CM only group but not in the CM + SUD group could reflect that enhanced salience processing to facial expressions of happiness and fear might indicate an adaptive mechanism, characterized by greater attention towards relevant social stimuli. In addition, the fact that in the CM only group, vmPFC was also more activated during the task, and had decreased connectivity to AI at rest, might indicate modulatory effects of vmPFC on salience processing. Previous evidence in PTSD patients supports our hypothesis, showing that increased activity in AI, aMCC and vmPFC during an emotional reactivity task was associated with improved symptomatology after exposure therapy treatment [44]. Finally, the finding of similar brain activity in the CM only and SUD groups suggests that the identified processes might primarily be protective in CM exposed individuals, who would otherwise be rendered vulnerable to SUD through an internalizing pathway, i.e. one that is driven by negative emotionality. In contrast, among individuals with SUD only, externalizing traits, i.e. impaired top-down control of incentive salience and reward-seeking behavior, may be the dominant category of vulnerability factors. A potential implication is that the characteristics found in the CM only, resilient group in our study do not to protect against this type of SUD risk.

We used the emotional conflict task to probe emotion regulation processes [25]. Here, the Stroop-like nature of incongruent trials robustly activated regions engaged by conflict processing and emotional interference [45], indicating increased cognitive load. Behaviourally, we replicated the typical interference triggered by concurrent conflicting information across all groups. However, we did not find behavioral effects associated with the original concepts of conflict monitoring or adaptation [25]. Recent work on test-retest reliability of the emotional conflict task shows good reliability for the typical Stroop-like effect of incongruent trials but only moderate or poor for conflict monitoring or resolution [46]. Finally, we identified a strong effect of emotion in congruent trials. Participants were significantly slower when presented with fearful compared to happy faces.

We found that the CM only group also had increased levels of the peripheral eCB AEA. AEA is proposed to function as a stress buffer [10] and in healthy adults, elevated AEA is associated with reduced stress reactivity and enhanced emotion regulation abilities [27, 29]. Although our findings are in general agreement with those reports, we did not find a main effect of stress to increase AEA levels using our experimental design. This apparent discrepancy may be related to differences in study design. Participants in the prior studies that found stress to increase AEA [27, 29] completed stress and control tasks on separate days, and the stress-induced increase in AEA was a between-session effect. In contrast, our study only involved a single session, and examined within-session stress-responses. This may limit the extent to which these results may be possible to compare. Evidence from preclinical models and human genetics suggests that elevated AEA is associated with corticolimbic connectivity that may facilitate emotion regulation [11, 12, 14]. Our findings are generally in line with these studies; as we find that the CM only group not only has higher AEA, but also has a unique neural activation pattern in key emotion-relevant regions previously shown to be associated with better treatment outcome [44]. Thus, the CM only group may constitute a subgroup of individuals with particularly high AEA levels, which, in turn, is protective against the type of processes otherwise making people vulnerable to developing SUD following CM exposure. Alternatively, CM exposure itself may result in increased AEA levels specifically within these individuals. Unfortunately, our cross-sectional approach precludes us from determining if high AEA levels were inherent to these individuals or a consequence of CM exposure. Regardless, these findings have important clinical implications, as pharmacological elevation of AEA has been proposed as a novel pharmacotherapeutic for trauma-exposed individuals and is currently being tested in clinical trials (EudraCT 2020-001965-36) [9, 47].

The prospective assessment of CM exposure allowed us to objectively discriminate between the SUD only and the CM + SUD groups, which would not have been possible to do with sufficient reliability using retrospective assessment. In fact, retrospectively self-reported CM was similar in all participants except the controls, supporting evidence of poor agreement between prospective and retrospective assessments [23]. Longitudinal evidence from large cohort studies highlights the poor within-subject reliability of subjective reports [48] and the crucial influence that psychopathology has on retrospective reports of CM [24]. Accordingly, we recently found in a sample which includes the participants examined in the current study, that CTQ scores show excellent discrimination of severe CM from healthy controls with no recorded CM, but no better than chance-level discrimination for individuals with SUD exposed or unexposed to CM [49]. These findings, point to the importance of acknowledging the impact of CM assessment method on reported findings and group categorization.

The main limitation of our study is the lack of stratification by type of CM and age at exposure, factors that have been shown to potentially contribute to inconsistencies in the literature [41]. Medical records indicate that those included in our sample had mainly been exposed to sexual or physical abuse, or both; and in addition, some had also been exposed to physical neglect [2]. Age at first exposure is not always clearly indicated in the records and sometimes several months or even years may have passed between age at first CM and contact with the CAP treatment unit. Another possible limitation is that CM included in this study are the most severe cases, given that a large proportion of those maltreated during childhood will not come to the attention of child protective services. This may limit generalizability of findings to the less severe end of the CM spectrum.

In sum, we identified possible mechanisms for resilience to developing SUD following CM, related to increased AEA levels and increased activity in salience and emotion regulation regions of the brain. Our results underscore the importance of assessing CM history for understanding the heterogeneity in the pathophysiology of SUD, as well as provide compelling additional support to eCB system modulation as a candidate therapeutic target [50]. Finally, an important direction for future research is exploring whether pharmacological treatments that target the eCB system may help to *prevent* the onset of SUD in at-risk individuals.

### Supplementary information

Supplemental material
