# Supplementary material for: Resilience to substance use disorder following childhood maltreatment: association with peripheral biomarkers of endocannabinoid function and neural indices of emotion regulation
Source: Mol Psychiatry. 2023 Apr 12;28(6):2563–71. doi: 10.1038/s41380-023-02033-y (PMC10611562; doi:10.1038/s41380-023-02033-y)
Supplement: Supplementary file 1 — Supplemental material [file 41380_2023_2033_MOESM1_ESM.docx]

**Supplementary Information**

Perini I, Mayo LM, et al. Resilience to substance use disorder following childhood maltreatment: evidence from central and peripheral measures

**Materials and Methods**

**Participants**

A first screening of medical records verified prospectively documented CM exposure, presence or absence of lifetime SUD diagnosis or contact with SUD clinics. Those who had no CM, had emigrated, deceased, lacked contact details, had current or life-time schizophrenia, bipolar or psychotic disorder, organic brain disorder, current suicidality, or cognitive impairment were excluded at this first selection. Medication use was assessed with self-reports and/or medical records. Participants prescribed psychotropic medications were included provided stable use for at least three months (Table 1). Participants prescribed central stimulants or melatonin omitted medications during the day and the evening before the experimental sessions. A study flow-chart is presented in Figure S1.

**Screening session**

During this session, participants underwent a psychiatric clinical assessment by a trained research nurse or study physician, and a structured Mini International Neuropsychiatric Interview (MINI-7),^1^ for DSM-5,^2^ Swedish version.^3^ Lifetime SUD was identified using the regional health care register and contact with addiction clinics, and current SUD was assessed using the MINI, self-reported current problems and urine drug screens. Controls were at this point excluded if CM was identified in their medical records, during clinical assessment or with the MINI-7 post-traumatic stress disorder (PTSD) module. After determining eligibility, participants received research information, provided written informed consent, and completed self-report questionnaires. Questionnaires assessed self-reported CM, using the Childhood Trauma Questionnaire-Short Form (CTQ).^4,5^ Current alcohol and drug use severity were assessed using the Alcohol Use Disorders Identification Test (AUDIT^6^) and the Drug Use Disorders Identification Test (DUDIT^7^). Emotion regulation and personality traits were assessed using the Difficulties in Emotion Regulation Scale (DERS-16)^8^ and NEO Five-Factor Inventory (NEO-FFI-3)^9^ questionnaires.

**Questionnaires**

The 28-item version, validated in Sweden^10^ of the Childhood Trauma Questionnaire-Short Form (CTQ), was used, with 25 clinical items equally distributed across five subscales including emotional abuse, emotional neglect, sexual abuse, physical neglect and physical abuse.^4,5^ Items are rated 1 (never true) to 5 (very often true), generating a total CTQ-score between 25 and 125, and subscale scores ranging between 5 and 25. Three additional items assess minimization/denial. The NEO Five-Factor Inventory (NEO-FFI-3) assesses personality traits according to the Five Factor Model as described by McCrae and Costa^9^ : Neuroticism, Extraversion, Openness, Agreeableness and Conscientiousness. The form consists of 60 items, 12 for each personality trait. Trait scores were converted into sex-specific standardized T-scores with a mean of 50 and a standard deviation of 10 before analysis, based on Swedish normative data.^11^ The AUDIT^6^ is a well-established, 10–item questionnaire measuring alcohol use problems (total score range 0–40). Scores between 0–7 indicate low-risk alcohol use, whereas scores of 8+ indicate hazardous drinking. The DUDIT^7^ is a 11-item questionnaire measuring drug use problems (total score range 0–44). For males, scores of 6+, and for females, scores of 2+ indicate problematic drug use. The DERS-16 is a self-report questionnaire (short form) that addressed the ability to identify and regulate emotions. The CPRS^8,12^ is a 19-item self-report version covering depression, anxiety and obsessional symptoms. The CPRS comprises the Montgomery-Asberg Depression Rating Scale and the Brief Anxiety Scale, which are both subscales drawn from the CPRS plus two questions about obsessional symptoms.

**Behavioral Session**

*Facial electromyography (EMG) and electrocardiography (ECG)*

Facial EMG sensors consisted of 4mm silver/silver chloride electrodes filled with electrode gel; two placed on each muscle location to form bipolar recording pairs. Recording electrodes were placed on muscles on the left side of the face and an 8mm ground electrode was placed on the forehead near the hairline. Facial EMG sensors were placed over the *zygomaticus major* (“zygomatic”; cheek), *corrugator supercilli* (“corrugator”; above the eyebrow), and *orbicularis oculi* (“orbicularis”; below the eye) muscles. Sites were cleaned with alcohol and lightly abraded and any site with impedance over 20kΩ (measured with a Model 1089 MK III Checktrode; UFI, Morro Bay, CA, USA) was reapplied. EMG signals were amplified, filtered through a 10-500 Hz band pass and 50 Hz comb band stop filter, digitized at 1 kHz, re-filtered, rectified, and integrated over 20ms using EMG100C amplifiers, MP150 Data Acquisition system and Acknowledge software from Biopac Systems (Biopac Systems, Inc, Camino Goleta, CA, USA). In all tasks, trials with excessive baseline activity or artefactual activations were identified and excluded by trained, blinded raters. The number of trials excluded based on these factors ranged from 2 to 15% across muscle location (zygomatic, corrugator, orbicularis) and task. For the affective image task, facial EMG responses for the corrugator and zygomatic muscles was assessed through the task, quantified as the mean EMG amplitude during the 6sec image presentation compared to the preceding 1 sec baseline.

Electrocardiography (ECG) was assessed via disposable Ag/AgCl snap gel electrodes placed at the right supraclavicular fossa and mid-axillary on the left side of the abdomen. Sites were cleaned with alcohol prior to electrode placement. Data were relayed to the Biopac ECG100C amplifier, filtered, and digitized at 1 kHz. Electrodermal activity (EDA) was assessed via two disposable Ag/AgCl contact snap electrodes (EL507; Biopac) pre-gelled with isotonic (*.*5% chloride salt) gel placed on the thenar and hypothenar of the right hand. Data were relayed to a Biopac EDA100C amplifier, employing a constant voltage technique and sampling the absolute, direct current skin conductance at the rate of 20 samples per second. Non-specific skin conductance responses (SCRs) were obtained with an additional high pass filter applied to the EDA channel to remove the slow changes in skin conductance level (SCL). SCR threshold was set at *.*2µs and responses with an amplitude of less than 10% of the mean were disregarded. SCL and SCR frequency were collected during the 10min task period and compared to a 5min baseline immediately preceding the task.

### *Endocannabinoid analysis*

The lipid concentrations were analysed using a liquid chromatography tandem mass spectrometry (LC-MS/MS) method based on a previously published method.^13^ Before the measurements, lipids were extracted from plasma following a previously described protocol.^14^ All standards (Arachidonylethanolamide, AEA; 2-arachidonoylglycerol, 2-AG; Palmitoylethanolamide, PEA; Oleoyl ethanolamide, OEA), and internal standards (Arachidonylethanolamide-d4, AEA-d4; 2-arachidonoylglycerol-d5, 2-AG-d5; Palmitoylethanolamide-d4, PEA-d4; Oleoyl ethanolamide-d4, OEA-d4) were purchased from Cayman Chemicals (Ann Arbor, MI, USA). Briefly, plasma samples were vortexed after being thawed on ice and 1.2 mL 100% acetonitrile (ACN; Sigma Aldrich, USA) was added to 300 µL of the sample and vortexed. 30 µL of deuterated internal standard [AEA-d4, OEA-d4, PEA-d4 (50 nM)) and 2AG-d5 (1000nM); Biotage, Sweden] was added to each plasma and blank sample, before centrifugation (5 min, 3000g, 4^o^ C). The supernatant was transported to 4.5 mL MilliQ-H_2_O with *.*133% triflouro acetic acid (TFA; Sigma Aldrich, USA). Thereafter the samples were transferred to C8 Octyl SPE columns (6 mL, 200mg; Biotage, Sweden). Prior to transferring the samples, the C8 Octyl SPE columns were activated with 1ml Methanol (Merck, Darmstadt, Germany) and washed with 1mL MilliQ-H_2_O using a Biotage ® Pressure+ 48 machine. After the samples were added, the columns were washed with ACN (20% with *.*1% TFA) and samples were eluted with ACN (80% with *.*1% TFA). The eluates were evaporated to dryness in a SpeedVacc (Thermo Fisher, Ann Arbor, MI, USA) and stored in -80 °C until analysis. On the day of the analysis, the samples were reconstituted in 30µl Mobile phase A (methanol-milliQ water-acetonitrile (4/4/2) (v/v/v) with *.*1 % (v/v) formic acid and 1g/L ammonium acetate), then vortexed and transferred into glass vials designed for the LC-MS/MS. The injection volume was 10 μL. We used an LC-MS/MS system consisting of a Thermo Scientific Accela AS auto sampler and Accela 1250 pump coupled to a Thermo Scientific TSQ Quantum Access max triple quadrupole mass spectrometer with a HESI II probe as ionization source. LC was performed using gradient elution with mobile phase A, and mobile phase B (containing methanol-ACN (7/3) (v/v) with *.*1% (v/v) formic acid and 1g/L ammonium acetate). The gradient elution was applied with a constant flow of 250 μL/min. We started with 100% mobile phase A during the first 1.5 min and followed this using a linear increase towards 100% mobile phase B, which was achieved after 9 min in total. Between the 11th and 12th min the gradient changed linear to 100% mobile phase A, which was maintained for 1 min. An Xbridge C8 analytical column (2.1 mm × 150 mm) with the particle size 2.5 µm obtained from Waters (Dublin, Ireland) was used. We used the following selected reaction monitoring (SRM) (m/z) transitions: 348.3/ 62.4; 326.3/62.4; 300.3/62.4; and 379.3/287.3 for AEA, OEA, PEA, and 2-AG, respectively. For the corresponding internal standards, we used the following transitions: 352.3/ 62.4; 330.3/62.4; 304.3/62.4; and 384.3/287.3 for AEA-d4, OEA-d4, PEA-d4, and 2-AG-d5, respectively. The linearity of the measuring ranges was assessed with standard curves ranging from 1-25 nM for AEA and 10-500 nM for OEA, and PEA, and 50-1250 nM for 2-AG in duplicate. The linearity of the standard curves was R^2^ ≥ *.*96 for all analytes. Isotopic dilution was used for quantification of the analytes, performed according to their area ratio of their corresponding deuterated internal standard signal area. 2-AG was quantified as the sum of 1- and 2-AG, which has been reported by others.^15,16^ Linear regression and X^2^ weighting were applied. Undetected levels were considered as 0 nM. Xcalibur® (version 2.1, Thermo Scientific) software was used for peak integration and quantification.

Endocannabinoid values were log transformed due to non-normality of the distribution and analyzed using ANOVAs. Some 2-AG values were below the limit of detection by mass spectroscopy, resulting in missing data. As a consequence, only baseline values for 2-AG are included in subsequent analyses.

### *Cortisol analysis*

Cortisol levels were obtained from plasma using the DetectX Cortisol Enzyme Immunoassay kit (Arbor Assays; Ann Arbor, MI, USA) according to manufacturer instructions. Cortisol values were log transformed due to non-normality; these transformed values were used in all subsequent analyses. Baseline differences in plasma cortisol were analyzed as the dependent variable in a one-way ANOVA. Cortisol responses to stress were analyzed using a RM-ANOVA with time as a within-subjects factor.

### *Genotyping*

At the end of the screening visit and upon participant inclusion, blood samples were collected for genotyping in 6mL EDTA tubes (BD, Franklin Lakes, NJ USA). Blood samples were stored in -80^o^ C until analysis. DNA was extracted using QIAamp DNA Blood Maxi Kit (Qiagen, Hilden, Germany) and analyzed on the Illumina Global Screening Array-Multi Disease version3 (GSA-MDv3) (Illumina, San Diego, California, USA). Genotyping was performed by the SNP&SEQ Technology Platform in Uppsala (www.genotyping.se). Here, we specifically looked at the *FAAH C385A* (rs324420) allele frequency between groups. The facility is part of the National Genomics Infrastructure supported by the Swedish Research Council for Infrastructures and Science for Life Laboratory, Sweden.

### *Affective Images*

The affective image task^17^ was completed before and after the stress task. Positive, neutral, and negative images were selected from the International Affective Picture System (IAPS^18^) and divided into two separate sets matched on normative ratings of valence and arousal and the presence or absence of social content.^18-20^ Each task contained 12 images of each category (positive, neutral, negative). Participants viewed a single image for 6 sec and then rated valence from -4 (negative) to +4 (positive) and physiological arousal on a scale of 0 to 9. Facial EMG responses were quantified as the mean EMG amplitude during the 6 sec image presentation compared to the preceding 1 sec baseline. Affective responses to emotional stimuli were assessed using separate RM-ANOVA with stimulus type (positive, neutral, negative) as the within-subject factor for each muscle (corrugator, zygomatic) and self-report rating (valence, arousal).

*Maastricht Acute Stress Test*

The Maastricht Acute Stress Test (MAST), a modified version of the classic cold pressor task, is a quick and noninvasive approach to elicit robust autonomic and glucocorticoid stress response.^21^ The 10 min task consisting of alternating “hand immersion” (HI) trials and “mental arithmetic” (MA) trials. In HI trials, participants placed their left hand in cold water (1-4 degrees Celsius) for up to 90 sec. In MA trials, participants performed mental math aloud, with mistakes resulting in negative feedback (e.g. “start over”) and successful attempts resulting in prompting to increase speed. Upon completion of the task, participants completed a questionnaire assessing how “unpleasant,” “painful,” “stressful,” and “boring” the task was using a 100 mm visual analog scale. In addition, self-reported positive and negative affect before and after the stress and control tasks was assessed via the state version of the Positive and Negative Affect Schedule (PANAS-S). Blood samples were collected via an indwelling catheter in the arm not submerged during the task. Samples were collected immediately before and after the task, as well as after 15 and 30 min of recovery. Participants who were unable to have an intravenous catheter successfully inserted or lost patency throughout one or both sessions were eliminated from biochemical analysis. Changes in physiological variable (EDA, heart rate) and subjective stress responses were assessed using a separate RM-ANOVA with time (baseline, stress) as a within-subjects factor.

**Magnetic Resonance Imaging session**

*Emotional Conflict Task*

We used two linear mixed effects (LME) models that considered the full-factorial nature of group recruitment strategy and included CM and SUD as factors. In the first model, behavioral responses were categorized in two separate within-subject factors, depending on current and previous trial type. Data were entered in a 2x2x2x2 LME analysis, with factors: CM (yes/no) X SUD (yes/no) X current (congruent/incongruent) X previous (congruent/incongruent).

*Negative-affect picture matching task*

The task was modified from the original task by Hariri et al..^22^ During each trial, participants were presented with three pictures and were instructed to select which of the pictures on the bottom was equal to the one on top, by pressing with index or middle fingers on a button pad positioned on the right hand. Three picture categories were used: angry and fearful facial expressions taken from the Karolinska Directed Emotional Faces Catalogue,^23^ negative valence pictures (arousal=6.5 valence=3.2) from the International Affective Picture System (IAPS) catalogue,^24^ and geometric shapes as controls. Blocks of 6 trials presented consecutively, with a between-block inter stimulus interval of 14000 ms, were presented 2 times per picture category. Before each block a 3000 ms instruction text indicated what category was going to be presented. There were two runs in total. Five regressors were included in the analysis: three regressors modelled each 2000 ms trial according to picture category (face, IAPS, shapes). The remaining two regressors modelled the instruction text and modelled general motor response, corresponding to any event in which the participant pressed a button. A 2x2x2 LME analysis was performed with the following factors: CM (yes/no) X SUD (yes/no) X trial (face/IAPS). Face and IAPS levels reflect beta coefficients for the within-subject contrasts face-shape and IAPS-shape. Beta coefficients from significant interactions were compared between groups using One-Way ANOVAs and post-hoc comparisons were corrected with Tukey’s test.

*MRI data Preprocessing and Analysis*

Anatomical and functional blood oxygen-level-dependent (BOLD) data were collected on a Siemens MAGNETOM Prisma 3T MRI scanner (Siemens healthcare AB, Stockholm, Sweden) equipped with a 64-channel head coil. Functional data was collected using an echoplanar imaging (EPI) sequence: TR=901 ms; TE=30 ms; flip angle=63°; field-of-view (a>p) x (r>l) x (f<h)=192 mm x 192 mm x 144 mm; voxel size=3,0 mm isotropic; no slice gap; number of axial slices (angled with the (anterior through posterior commissure (AC-PC) line)=48; number of volumes for emotional conflict task=800, number of volumes for resting state=800, number of volumes for negative affect picture task=320. Anatomical data was collected via T1-weighted imaging: TR=2300 ms; TE=2,36 ms; flip angle=8°; field-of-view (a>p) x (r>l) x (f<h)=263 mm x 350 mm x 350 mm; voxel resolution=0,9 mm isotropic; no slice gap; plane: sagittal; number of sagittal slices=208. EPI images were de-spiked, slice-time corrected, smoothed (4 mm) and motion-corrected. Each EPI volume was registered to the volume with the minimum outlier fraction, for motion correction and co-registration purposes. BOLD images were then warped to the MNI152_2009_SSW template space using linear and non-linear transformations via AFNI’s @SSwarper function. Nuisance effects due to head motion were accounted for by adding the motion parameters and their derivatives as regressors of no interest in the main regression. A combination of thresholds for motion censoring set at max 3.0 mm per TR, and outlier fraction set at *.*05, was applied, so that volumes exceeding these values were not included in the time-series regression. No between-group difference in motor censoring was observed for task-based or resting-state data (*ps*>*.*3). BOLD time-series data were deconvolved using the 3dDeconvolve function. For resting state data, the preprocessing steps were the same as for the task-based preprocessing, except that a freesurfer-based parcellation was first performed on T1-weighted data using the function recon-all, imported to AFNI using @SUMA-Make_Spec_FS and subsequently used for tissue-based regression. In addition, the covarying signals between gray and white matter regions were removed using the data driven APPLECOR method.^25^

Results were thresholded at a per-voxel *P=.*002, and multiple comparison corrected at *alpha*=*.*05.^26^ Cluster size threshold for multiple comparison correction was estimated by entering spatial smoothness parameters of the residuals in the 3dClustsim simulation function. As simulation mask, we used a gray matter mask that included the union of 80% of the subjects’ EPI-masks surviving censoring.

**Results**

**Behavioral Session**

### *Affective images*

We found no differences in valence ratings at baseline (Figure S2). As expected, there was a main effect of picture type (F_2,194=_1192, *P<.*001, partial η^2^=.92) on ratings of valence, but no other effects or interactions. There was a significant stress x picture type interaction (F_2,190_= 4.08, *P=.*018, partial η^2^=.079), which was specific to negative images (F_3,95_=3.21, *P=.*026, partial η^2^=.092). In particular, following stress exposure, the CM only group rated negative images as less negative as compared to the SUD only group (*P=.*026).

We found no significant group differences in facial EMG responses to emotional images at baseline or in response to stress. At baseline, there as a main effect of picture type (F_2,194_=47.9, *P<.*001, partial η^2^=.33) on corrugator reactivity, such that negative pictures resulted in increased corrugator activity and positive images elicited a reduction in corrugator activity. There were no other main effects or interactions, nor was there a significant effect of stress. At baseline, there was a main effect of picture type on zygomatic activity (F_2,192_=28.0, *P<.*001, partial η^2^=.23) and an interaction with picture type and CM exposure (F_2,192_=3.75, *P=.*025, partial η^2^=.038), however post hoc tests failed to uncover significant pair-wise effects. There were no other significant main effects or interactions, nor was there a significant effect of stress.

### *Stress*

Overall, there were limited differences in stress reactivity across groups. As expected, there was a main effect of stress on non-specific skin conductance response (SCR) levels (F_1,91_=74.9, *P<.*001, partial η^2^=.45; Fig. 2B), as well as a between-subject effect of SUD on SCR levels (F_1,91_=6.30, *P=.*014, partial η^2^=.065) such that both SUD+ groups had lower SCR frequency irrespective of stress. There was also a main effect of stress on skin conductance level (SCL; F_1,90_=28.6, *P<.*001, partial η^2^=.25) and heart rate (F_1,91_=4.75, *P=.*032, partial η^2^=.050) but no interactions or between-group effects.

Participants rated the stress task on several measures, including arousal, which differed between groups (F_3,93_=3.04, *P=.*033, partial η^2^=.089), with a main effect of CM (F_1,93_=7.58, *P=.*007, partial η^2^=.075). Post hoc follow-up tests revealed a marginally lower arousal rating in the CM only group (*P=.*053) and CM+SUD (*P=.*089) compared to controls, but these did not reach significance. We found no differences in self-reported ratings of the stress task regarding how stressful (*P=.*80), unpleasant (*P=.*19), or painful (*P=.*84) the task was perceived.

The groups differed in self-reported negative affect (i.e., the PANAS) at baseline (F_3,96_=3.63, *P=.*015, partial η^2^=.10), including a main effect of CM (F_1,96_=9.99, *P=.*002, partial η^2^=.094) such that negative affect was significantly higher in the CM only group (*P=.*028) and marginally higher in the CM+SUD group (*P=.*087), as compared to controls. Baseline positive affect did not differ between groups (*P=.*87). Following stress exposure, positive affect decreased (F_1,95_=4.17, *P=.*044, partial η^2^=.042), but there were no differences between groups. Stress also increased self-reported negative affect (F_1, 95_=11.7, *P<.*001), but there were no interactions with group status.

**Magnetic Resonance Imaging session**

*Emotional Conflict Task*

Behavioral findings. The first 2x2x2x2 LME analysis, with factors: CM (yes/no) X SUD (yes/no) X current (congruent/incongruent) X previous (congruent/incongruent), showed no between-group effects. A robust main effect of current trial was found on RTs, F_1,254=_265, *P<*.001, such that RTs were significantly slower when the current trial was incongruent compared to congruent (mean difference=37 ms ± SEM 2 ms). In addition, the LME results revealed a separate main effect of previous trial, F_1,254=_7, *P=*.008, showing significantly slower RTs when the previous trial was incongruent compared to congruent (mean difference=6 ms ± SEM 2 ms), but there was no interaction effect between previous trial and current trial (*P=*.091). The accuracy results replicated the main effect of current trial, F_1,185_=109, *P*<.001, with significantly better performance when the current trial was congruent compared to incongruent (mean difference=5.75 % ± SEM *.*55 %). Previous trial showed no significant main effect on accuracy (*P=*.425), and there was no interaction with current trial (*P*=.164).

We also found a main effect of SUD (F_1,84=_5.86, *P*=.018), with significantly poorer performance amongst participants with SUD compared to those without (mean difference=-4.66% ± SEM 1.82%).

The second 2x2x2x2 LME analysis, with factors: CM (yes/no) X SUD (yes/no) X trial (congruent/incongruent) X emotion (fear/happy) showed similar results (Figure S2). Starting with reaction times (RT), the results of the second LME analysis showed significant main effects of trial, F_1, 235_=158, *P<*.001, and emotion, F_1, 235_=19, *P*<.001, as well as a trial x emotion interaction, F_1, 235_=6.73, *P=*.010. RTs were significantly slower on incongruent trials compared to congruent (mean difference=36.76 ms ± SEM 2.92 ms, *P<*.001), and slower in response to fearful faces compared to happy faces (mean difference=12.77 ms ± SEM 2.92 ms, *P<*.001). The interaction revealed that RTs were significantly slower in response to trials when words were congruent with fearful faces compared to trials with words congruent with happy faces (mean difference=20.34 ms ± SEM 4.30, *P<*.001). There was no difference between fearful and happy incongruent faces (*P=*.191). There was also a marginally significant main effect of SUD (F_1,87_=3.98, *P=*.049), showing that participants with SUD had slightly slower RTs overall compared to those with no SUD (mean difference=24.29 ms ± SEM 12.17 ms). For accuracy, we found a significant main effect of trial (F_1,223=_72, *P*<.001), and a separate main effect of SUD (F_1,84=_6.73, *P=*.011. Accuracy was significantly poorer on incongruent compared to congruent trials (mean difference=-5.54 % ± SEM *.*66 %, *P<*.001). Participants with SUD performed significantly worse compared to those without (mean difference=-4.02 % ± SEM 1.55 %, *P=*.011).

*Negative-affect picture processing*

One participant from the SUD group was excluded for excessive motion (Table1). A main effect of trial revealed significantly increased activity in several regions including bilateral insular cortex, bilateral midcingulate cortex and bilateral amygdala to face pictures, and increased activity in bilateral fusiform gyrus, posterior cingulate cortex/precuneus and vmPFC to IAPS pictures (Table S2).

The 2x2x2 LME analysis revealed a significant CMXSUD interaction in the caudal portion of the inferior parietal lobule (PGp, MNI=40, -65, 16; 14 voxels), in the fusiform gyrus (FG3, MNI=-26, -44, -20; 11 voxels), and lateral occipital cortex (V4t, MNI=52,-71,-1, 10 voxels). Post-hoc analysis revealed a trend-level group effect in PGp to faces (F*_3,88_*=2.46, *P=*.068 partial η^2^=.077) and IAPS (F*_3,88_*=2.48, *P=*.066 partial η^2^=.078), driven by increased PGp activity in CM only versus controls for both face and IAPS pictures (mean difference=*.*14, *P=*.041; mean difference=*.*14, *P=*.044).

**FIGURES**

**Figure S1.** CONSORT flow-chart of study participants. ^1^ Emotional Conflict Task; ^2^ Negative Affect Picture Task; ^3^ Resting State.

**Figure S2.** Baseline ratings of affective images.

Participants rated positive, neural, and negative affective images on the orthogonal measures of valence (left) and arousal (right). There were no group differences in valence ratings. However, the CM only group rated negative images as less arousing than control participants. * *P<.*05.


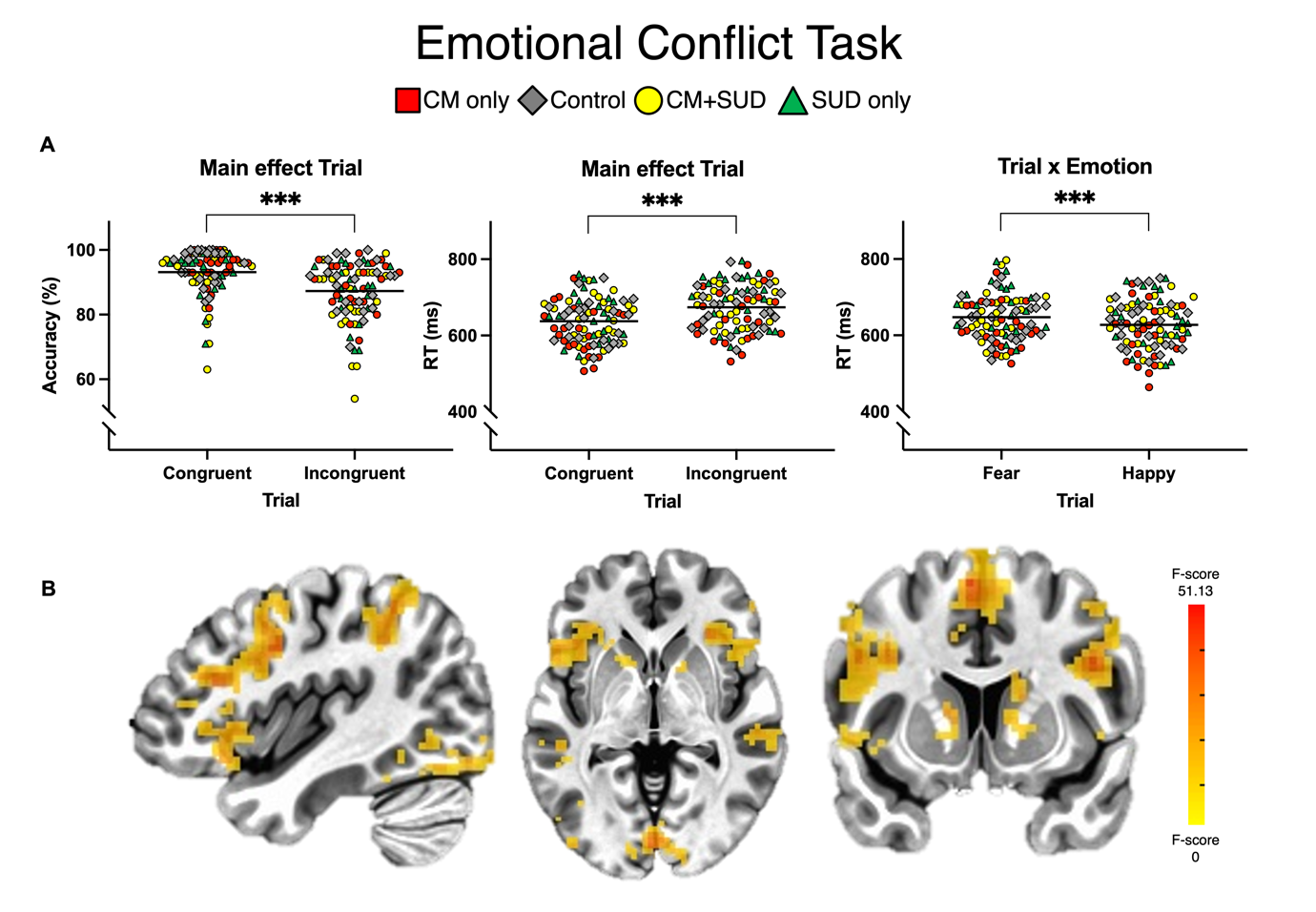


**Figure S3**. Emotional conflict task. 2x2x2x2 linear LME model was performed on behavioral and brain measures with factors: CM (yes/no) X SUD (yes/no) X Trial (congruent/incongruent) X Emotion (fear/happy). **(A)** Behavioral results. **(B)** Activation maps indicate increased activity to incongruent trials. Per voxel *P=.*002, multiple comparison corrected at alpha=*.*05. Left is left. *** *P<.*001.

**Tables**

| **Location** | **MNI coordinates (x, y, z)** | | | **#voxels** |
| --- | --- | --- | --- | --- |
| Inferior Frontal Gyrus, p. Triangularis (Area 45) | -50 | 19 | 22 | 885 |
| Inferior Frontal Gyrus, p. Opercularis (Area 44) | 43 | 10 | 28 | 625 |
| Intra Parietal Lobule (Area hIP2, IPS) | -47 | -44 | 46 | 586 |
| Superior Frontal Gyrus, Supplementary Motor Area | -5 | 10 | 58 | 410 |
| Intra Parietal Lobule (Area hIP3, IPS) | 38 | -53 | 53 | 374 |
| Inferior Temporal Gyrus (Area FG2) | 49 | -59 | -17 | 300 |
| Calcarine Gyrus, Primary Visual | 1 | -86 | 1 | 282 |
| Inferior Occipital Gyrus (Area h0c41la) | -44 | -80 | -14 | 265 |
| Temporo-Parietal-Occipital Junction (Area PGa IPL) | 58 | -50 | 10 | 103 |
| Posterior Superior Temporal Sulcus | -53 | -35 | 2 | 102 |
| Precentral Gyrus, Area 55b | 43 | 7 | 49 | 72 |
| Caudate | 16 | 10 | 13 | 67 |
| Cerebellum, Crus 2 | -14 | -77 | -35 | 56 |
| Supramarginal Gyrus (Area PFm, IPL) | 58 | -41 | 25 | 46 |
| Calcarine Gyrus, Primary Visual | 22 | -62 | 4 | 36 |
| Caudate | -14 | 11 | 8 | 25 |
| Cerebellum, Crus 2 | -26 | -80 | -44 | 24 |
| Caudate | -17 | -8 | 19 | 18 |
| Middle Occipital Gyrus, (Area h0c4lp) | -29 | -89 | 7 | 16 |
| Temporo-Parietal-Occipital Junction (Area PGcm IPL) | -47 | -47 | 16 | 16 |
| Precuneus (Area 7A, SPL) | 8 | -65 | 56 | 15 |
| Supramarginal Gyrus (Area PFm, IPL) | -65 | -47 | 34 | 14 |
| Calcarine Gyrus, Primary Visual (Area h0c2, V2) | 8 | -95 | 11 | 12 |
| Thalamus | -8 | -17 | 17 | 12 |
| Superior Medial Gyrus, Supplementary Motor Area | 13 | 25 | 64 | 12 |
| Cerebellum, Vermis 4/5 (Lobule V) | 4 | -53 | -23 | 11 |

**Table S1.** Emotional conflict task. Significant clusters for the main effect of trial following a 2x2x2X2 LME analysis with factors: CM (yes/no) X SUD (yes/no) X trial (congruent/incongruent) X emotion (fear/happy). All clusters indicate increased activity to incongruent versus congruent trials. Whole brain, gray matter level, per-voxel *P*=*.*002, *alpha*<*.*05. Voxel size=3,0 mm isotropic.

| **Location** | **MNI coordinates (x, y, z)** | | | **#voxels** |
| --- | --- | --- | --- | --- |
| Cerebellum, Vermis 6 * | 7 | -74 | -20 | 2544 |
| Inferior Frontal Gyrus (Area_45) * | 52 | 19 | 28 | 2040 |
| Ventromedial Visual Area 3 (Area FG3) | -26 | -56 | -11 | 1376 |
| Ventromedial Visual Area 3 (Area FG3) | 28 | -59 | -11 | 1177 |
| Temporo-Parietal-Occipital Junction (Area PGa IPL) * | 59 | -47 | 17 | 570 |
| Dorsal Precentral Gyrus, Area 6, Frontal Eye Fields * | -32 | -8 | 55 | 375 |
| Intra Parietal Sulcus (Area hIP3 IPS)* | 37 | -53 | 43 | 363 |
| Parieto-Occipital-Sulcus Area 1 | -20 | -59 | 19 | 241 |
| Inferior Occipital Gyrus (Area h0c1la) | -50 | -74 | -5 | 205 |
| Ventromedial Prefrontal (Area s32) | -8 | 34 | -14 | 198 |
| Anterior Insula * | -32 | 22 | 7 | 196 |
| Cerebellum Crus 1 (Lobule VIIa Crus I) * | 40 | -50 | -32 | 185 |
| Posterior Cingulate, Area 23 | -5 | -41 | 37 | 178 |
| Parieto-Occipital-Sulcus Area 1 | 10 | -53 | 13 | 127 |
| Intra Parietal Sulcus (Area hIP3 IPS)* | -32 | -56 | 43 | 127 |
| Thalamus * | 14 | -14 | 8 | 125 |
| Temporo-Parietal-Occipital Junction (Area PGa IPL) * | -53 | -53 | 14 | 114 |
| Superior Frontal Gyrus, Area 8B | -23 | 40 | 52 | 85 |
| Inferior Frontal Gyrus, p. Orbitalis, area 47r | -38 | 37 | -11 | 75 |
| Amygdala * | 20 | -8 | -14 | 75 |
| Middle Orbital Gyrus, Area 11l * | 26 | 53 | -14 | 59 |
| Middle Frontal Gyrus, Area 9_46v * | -35 | 53 | 14 | 51 |
| Amygdala * | -23 | -8 | -14 | 47 |
| Thalamus * | -14 | -14 | 7 | 42 |
| Putamen * | 26 | -2 | 14 | 27 |
| Inferior Frontal Gyrus, p. Triangularis, area 46 * | -44 | 38 | 26 | 25 |
| Inferior Occipital Gyrus (Area h0c2 V2) * | 29 | -98 | -8 | 20 |
| Cerebellum, Lobule VIII * | 26 | -44 | -47 | 15 |
| Posterior Insula (Area OP3 VS) | 44 | -8 | 8 | 14 |
| Inferior Occipital Gyrus (Area h0c41p) * | 46 | -86 | -11 | 13 |
| Caudate Nucleus * | 13 | -5 | 16 | 13 |
| Caudate Nucleus * | -20 | 7 | 19 | 13 |
| Inferior Frontal Gyrus, p. Orbitalis, area 47m | 31 | 37 | -11 | 12 |
| Cuneus (Area h0c4d V3A) * | 19 | -77 | 34 | 11 |
| Cerebellum Lobule X * | -23 | -38 | -44 | 10 |

**Table S2.** Negative affect picture task. Significant clusters for the main effect of trial following a 2x2x2 LME analysis with factors: CM (yes/no) X SUD (yes/no) X trial (face-shape/IAPS-shape). *=clusters with increased activity to face versus IAPS pictures. Whole brain, gray matter level, per-voxel *P*=*.*002, *alpha*<*.*05. Voxel size=3,0 mm isotropic.

**References**

1. Sheehan DV, Lecrubier Y, Sheehan KH, et al. The Mini-International Neuropsychiatric Interview (M.I.N.I.): the development and validation of a structured diagnostic psychiatric interview for DSM-IV and ICD-10. *The Journal of clinical psychiatry*. 1998;59 Suppl 20:22-33;quiz 34-57.

2. American Psychiatric Association. *Diagnostic and Statistical Manual of Mental Disorders: DSM-5*. 5th ed. American Psychiatric Association; 2013.

3. Allgulander C, Nilsson B. Rikstäckande primärvårdsstudie: Var fjärde patient lider av ångest och depression. *Lakartidningen*. 2003;100:832-8.

4. Bernstein DP, Fink L, Handelsman L, et al. Initial reliability and validity of a new retrospective measure of child abuse and neglect. *Am J Psychiatry*. Aug 1994;151(8):1132-6. doi:10.1176/ajp.151.8.1132

5. Bernstein DP, Stein JA, Newcomb MD, et al. Development and validation of a brief screening version of the Childhood Trauma Questionnaire. *Child Abuse Negl*. Feb 2003;27(2):169-90. doi:10.1016/s0145-2134(02)00541-0

6. World Health O. AUDIT: the Alcohol Use Disorders Identification Test : guidelines for use in primary health care / Thomas F. Babor ... [et al.]. 2nd ed ed. Geneva: World Health Organization; 2001.

7. Berman AH, Bergman H, Palmstierna T, Schlyter F. Evaluation of the Drug Use Disorders Identification Test (DUDIT) in criminal justice and detoxification settings and in a Swedish population sample. *Eur Addict Res*. 2005;11(1):22-31. doi:10.1159/000081413

8. Bjureberg J, Ljótsson B, Tull MT, et al. Development and Validation of a Brief Version of the Difficulties in Emotion Regulation Scale: The DERS-16. *J Psychopathol Behav Assess*. Jun 2016;38(2):284-296. doi:10.1007/s10862-015-9514-x

9. Costa PT, McCrae RR. *Revised NEO personality inventory (NEO PI-R) and NEP five-factor inventory (NEO-FFI) : professional manual*. Psychological Assessment Resources; 1992:vi, 101 p.

10. Gerdner A, Allgulander C. Psychometric properties of the Swedish version of the Childhood Trauma Questionnaire—Short Form (CTQ-SF). *Nordic Journal of Psychiatry*. 2009/01/01 2009;63(2):160-170. doi:10.1080/08039480802514366

11. Kallmen H, Wennberg P, Bergman H. Psychometric properties and norm data of the Swedish version of the NEO-PI-R. *Nord J Psychiatry*. Oct 2011;65(5):311-4. doi:10.3109/08039488.2010.545433

12. Svanborg P, Asberg M. A new self-rating scale for depression and anxiety states based on the Comprehensive Psychopathological Rating Scale. *Acta psychiatrica Scandinavica*. Jan 1994;89(1):21-8.

13. Stensson N, Ghafouri N, Träff H, Anderson CD, Gerdle B, Ghafouri B. Identification of lipid mediators in peripheral human tissues using an integrative in vivo microdialysis approach. *Journal of Analytical and Bioanalytical Techniques*. 2016;7:306doi:10.4172/2155-9872.1000306

14. Stensson N, Ghafouri B, Gerdle B, Ghafouri N. Alterations of anti-inflammatory lipids in plasma from women with chronic widespread pain - a case control study. *Lipids Health Dis*. Jun 12 2017;16(1):112. doi:10.1186/s12944-017-0505-7

15. Balvers MGJ, Verhoeckx KCM, Witkamp RF. Development and validation of a quantitative method for the determination of 12 endocannabinoids and related compounds in human plasma using liquid chromatography–tandem mass spectrometry. *Journal of Chromatography B*. 2009;877(14-15):1583-1590 %@ 1570-0232.

16. Hill MN, Miller GE, Carrier EJ, Gorzalka BB, Hillard CJ. Circulating endocannabinoids and N-acyl ethanolamines are differentially regulated in major depression and following exposure to social stress. *Psychoneuroendocrinology*. 2009;34(8):1257-1262 %@ 0306-4530.

17. Mayo LM, Asratian A, Lindé J, et al. Protective effects of elevated anandamide on stress and fear-related behaviors: translational evidence from humans and mice. *Molecular psychiatry*. 2020;(1476-5578 (Electronic))

18. Lang PJ, Greenwald MK, Bradley MM, Hamm AO. Looking at pictures: affective, facial, visceral, and behavioral reactions. *Psychophysiology*. May 1993;30(3):261-73. doi:10.1111/j.1469-8986.1993.tb03352.x

19. Mayo LM, Perini I, Gustafsson PA, et al. Psychophysiological and Neural Support for Enhanced Emotional Reactivity in Female Adolescents With Nonsuicidal Self-injury. *Biol Psychiatry Cogn Neurosci Neuroimaging*. Nov 24 2020;doi:10.1016/j.bpsc.2020.11.004

20. Mayo LM, de Wit H. Acquisition of responses to a methamphetamine-associated cue in healthy humans: self-report, behavioral, and psychophysiological measures. *Neuropsychopharmacology*. Jun 2015;40(7):1734-41. doi:10.1038/npp.2015.21

21. Smeets T, Cornelisse S, Quaedflieg CWEM, Meyer T, Jelicic M, Merckelbach H. Introducing the Maastricht Acute Stress Test (MAST): a quick and non-invasive approach to elicit robust autonomic and glucocorticoid stress responses. *Psychoneuroendocrinology*. 2012;(1873-3360 (Electronic))

22. Hariri AR, Tessitore A, Mattay VS, Fera F, Weinberger DR. The amygdala response to emotional stimuli: a comparison of faces and scenes. *Neuroimage*. Sep 2002;17(1):317-23.

23. Goeleven E, De Raedt R, Leyman L, Verschuere B. The Karolinska Directed Emotional Faces: A validation study. *Cognition and Emotion*. 2008/09/01 2008;22(6):1094-1118. doi:10.1080/02699930701626582

24. Lang PJ, Bradley MM, Cuthbert BN. *International Affective Picture System (IAPS): Technical Manual and Affective Ratings.* Center for the Study of Emotion and Attention University of Florida: Gainesville, F.; 1997.

25. Marx M, Pauly KB, Chang C. A novel approach for global noise reduction in resting-state fMRI: APPLECOR. *Neuroimage*. Jan 1 2013;64:19-31. doi:10.1016/j.neuroimage.2012.09.040

26. Cox RW, Chen G, Glen DR, Reynolds RC, Taylor PA. FMRI Clustering in AFNI: False-Positive Rates Redux. *Brain connectivity*. Apr 2017;7(3):152-171. doi:10.1089/brain.2016.0475
